# Forty years after Alma-Ata: how people trust primary health care?

**DOI:** 10.1186/s12889-020-09082-w

**Published:** 2020-06-15

**Authors:** Homayoun Sadeghi Bazargani, Mohammad Saadati, Jafar Sadegh Tabrizi, Mostafa Farahbakhsh, Mina Golestani

**Affiliations:** 1 Research Center for Evidence Based Medicine, Tabriz University of Medical Sciences, Tabriz, Iran; 2 Road Traffic Injury Research Center, Tabriz University of Medical Sciences, Tabriz, Iran; 3 Tabriz Health Services Management Research Center, Tabriz University of Medical Sciences, Tabriz, Iran; 4 Psychiatrics Research Center, Tabriz University of Medical Sciences, Tabriz, Iran

**Keywords:** Primary health care, Trust, Public, Policy development

## Abstract

**Background:**

Primary Health Care (PHC) was introduced as the first level of health services delivery after Alma-Ata declaration. However, after forty years, it needs to be more trustful to achieve its predefined objectives. Public trust in PHC is one of the neglected issues in the context. The aim of this study is to evaluate public trust in PHC in Iran**.**

**Methods:**

The present investigation is a household survey conducted in East Azerbaijan Province, Iran. Two-stage cluster sampling method with Probability Proportional to Size (PPS) approach was used**.** Totally, 1178 households were enrolled in the study. PHC trust questionnaire and Ultra-short version of Socio-Economic Status assessment questionnaire (SES-Iran) was used for data collection. Data were analyzed using STATA software (version 15) through descriptive statistics and linear regression.

**Results:**

The mean ± SD age of the participants was 41.2 ± 15.1 and most (53.7%) were female. Mean score of PHC trust was 56.9 ± 24.7 (out of 100). It was significantly different between residents of Tabriz (the capital of province) and other cities in the province (*p* < 0.001). Linear regression showed that younger age, gender, insurance type, being married, and households higher socio-economic status had a significant positive effect on PHC trust level with R^2^ = 0.14383.

**Conclusions:**

Public trust in PHC system in Iran needs to be improved. Individual variables had a small but key role in trust level. PHC trust cannot be only affected by individual’s variables and experiences but also by health system and health providers’ characteristics and public context in which PHC system exists. PHC trust level could be used as a public indicator in health systems especially in Low and Middle Income Countries (LMIC) to contribute in system strengthening policies at the national and international levels.

## Background

Primary Health Care (PHC), as the first level in the delivery of health services, was extended after Alma-Ata Declaration, worldwide [1]. PHC was introduced as an approach to achieve “health for all” objective by providing proper, accessible, acceptable, and affordable services for most of the population in a country [2]. Efficient and responsive PHC strengthens integration of health services and improve health outcomes in a community [3, 4]. One of the most key determinants of health services effectiveness is trust [5]. Interpersonal and public trust in health are crucial issues with relation to the health system or providers and patients [6, 7]. Implementing patient-centered approach, shared decision making, and providing patients with information by health providers can contribute in patients more trust [8].

Trust in health system is as an indicator of public support and could be served as the orienting variable in health policy making [9]. A study of trust in health system in 33 countries revealed that the low level of public trust has originated from health system inability in promoting community health [10]. It was revealed that low level of trust in health providers or organizations lead to the underuse of vaccination and health services [11, 12]. Moreover, it caused delay in care-seeking and lower adherence to the instruction provided by health service providers [13–15]. However, according to the literature, trust is significantly associated with disease outcomes and patients’ well-being [16, 17]. In a comparative study, it was revealed that German people had the least level of trust in health system when compared with England, Wales and the Netherlands. The mean score of trust in health system was reported to be 7 and 5.3 out of 10 in the Netherlands and Iran, respectively [5, 9]. It is reported to be affected by different variables such as age, gender, education, religion, race, and socio-economic status [18–21]. Most of the previous studies on trust have focused on whole health system [9], health providers or organizations [22, 23], and health insurances [24]. Only some have taken a furtive glance on public trust in PHC. Using visual scaling, Tabrizi et al. (2016) reported that trust on health system was 10.83 out of 20 for PHC in Iran [5].

According to Gille et al. (2015) due to the role of trust/mistrust in health system, it is needed to provide more evidences to improve the efficiency of health systems [25]. Public trust has been used as a public indicator and has a key role in developing health policies therefore, it is necessary to be measured constantly using valid tools [26]. Measuring public trust in PHC provides evidences on people experiences and also the accountability and performance of PHC system. The present investigation in fact, tries to evaluate public trust in PHC system in East-Azerbaijan province in Iran.

## Methods

### Study design

A household survey was conducted in East-Azerbaijan province, Iran in 2015. It is a part of a larger project designed to assess the baseline and time trend of health variables affected by a PHC reform in Iran. Methodological details were published previously as the research [3] and intervention protocols [27].

### Study setting

Iran PHC system is one of the successful systems in the world with the outstanding achievements. Some of PHC performance indicators are as following: Infant Mortality Rate (IMR) per 1000 live births = 9.48, access to PHC in rural areas = 99%, Maternal Mortality Rate (MMR) per 100,000 live births = 19 and vaccine uptake rate = 99% in most of vaccination programs [28, 29]. PHC services are delivered through health houses in rural and health centers in urban areas. University of Medical Sciences in each province is the responsible governing body for health system. Due to active follow-up by health centers, nearly all of the households are in contact and familiar with PHC system and use its common free services. In urban areas, it is free to bypass PHC and contact hospital services but in rural, individuals should be referred through referral system [28, 29].

East Azerbaijan province with the total population of 3,900,000 people (based on 2016 census) is located in northwest of Iran. Tabriz as the capital city is the most developed, populated (1,773,033 people), and oldest city in the province. Along with city health centers, universities of medical sciences and provincial authorities are placed in capital cities of provinces, such as Tabriz, in Iran. Moreover, private and other sectors providing health services are mostly established in capital cities. In contrast, in other cities, majority of health services are provided by government [29, 30].

### Sampling

Sampling was done through two-stage cluster with Probability Proportional to Size (PPS) approach. One county was selected randomly as representative of each of five geographical parts (central, northeast, northwest, southeast and southwest) of the province.

Accordingly, we selected Tabriz metropolitan as the province capital, Oskou in central part, Varzeghan and Marand city in northeast and northwest, respectively and Bonab in southwest and Mianeh in southeast of the province.

In Tabriz city, 120 clusters comprising 20 households were allocated and the National Demographic Health Survey study (2011) was used as sampling framework. Similarly, 120 clusters were allocated to the other cities based on PPS. In cities other than Tabriz, the national population census was utilized as sampling framework.

### Questionnaires

PHC trust questionnaire was used for data collection [26]. The validity (Kappa coefficient = 0.94) and reliability (Cronbach-Alpha = 0.98, ICC = 0.94; CI: 0.87–0.97) of the scale was approved. It included 2 sections: first, demographic data such as age, gender, education, insurance, marital status and household dimension, and in the second section it covered 30 items on trust in PHC. Moreover, ultra-short version of Socio-Economic Status assessment questionnaire (SES-Iran) being validated in former studies was used for data collection [31, 32].

### Data collection and analysis

Based on the main plan for data collection [3], households number 6–10 (600 households) in each cluster, were asked to response the PHC trust questionnaire. Each household was approached for data for 3 times. Head of the households or housewife were interviewed by a trained questioner. In case of households’ head or housewife’s inability, an educated and informant member with age >15, was interviewed. Residency time ≥ 6 months was considered as inclusion criteria for the households. Descriptive statistics including frequency, mean ± and inferential statistics based on data normality were used for data analysis through STATA 15.

## Results

Totally, 1178 households were enrolled in the study. The mean age of the participants was 41.2, ±15.1 and most of them (53.7%) were female. Nearly a quarter of the participants (24.15%) had elementary education and 84.6% were married. The household size mean was 3.5 ± 1.2. participants’ demographics are presented in Table 1.

**Table 1:** Demographic characteristics of the study population

Variable		Tabriz Sample % (N)	Other cities Sample % (N)
Gender	Male	55.25 (352)	52.26 (335)
	Female	44.75 (285)	47.74 (306)
Education	Illiterate	17.49 (110)	21.63 (138)
	Elementary	22.1 (139)	26.18 (167)
	Under-diploma	23.21 (146)	22.26 (142)
	Diploma	23.69 (149)	18.5 (118)
	BSc	12.08 (76)	10.97 (70)
	MSc and upper	1.46 (9)	0.47 (3)
Insurance	No-insurance	14.75 (90)	4.04 (25)
	Health (public workers)	8.69 (53)	10.02 (62)
	Social security	59.51 (363)	43.62 (270)
	Military	2.62 (16)	2.26 (14)
	Rural	0.66 (4)	34.73 (215)
	Self-insured	0.49 (3)	0
	Iranian (public)	12.46 (76)	5.01 (31)
Marital status	Married	83.49 (521)	85.85 (546)
	Single	16.51 (103)	14.15 (90)

Approximately, 16% of the households had a member hospitalized at least once during the year before the study. Based on the participants’ responses; 6.65, 5.79, and 14.07% of the households had at least a member with diabetes, depression, and hypertension, respectively.

The mean score of the PHC trust was 56.9 ± 24.7 (out of 100). PHC trust score distribution was skewed based on Shapiro-Wilk W test (*p* < 0.05) and therefore, nonparametric analyses were used. Results revealed that the PHC trust score was different between inhabitants of Tabriz and other cities in the province (*p* < 0.001). Moreover, women had significantly more trust on PHC than men (Table 2). Individuals with higher education had lower level of trust when compared to those with diploma and under-diploma level of education but the difference was not significant.

**Table 2:** Relation between PHC trust score and participants characteristics

Variable		PHC trust score (Mean ± SD)	*P*-Value
Gender	Male	53.86 ± 25.7	0.001
	Female	59.53 ± 23.4	
Participants residential city	Tabriz inhabitants	49.32 ± 24.63	0.001
	Other cities inhabitants	64.13 ± 24.68	
Marital status	Married	57.81 ± 24.3	0.007
	Single	52.14 ± 26.03	

It was revealed that people with chronic disease (diabetes, hypertension, and depression) had a lower trust on PHC compared to others (*p* < 0.05). In opposite, people who suffered an injury from road accidents or falling, had more trust on PHC than others, however the difference was not statistically significant. In terms of households Socio-Economic Status (SES), the majority of households (89.2%) rated their economic capacity as low (SES score ≤ 25) and middle (25 < SES score ≤ 50). Trust level was significantly different between the households with different level of SES (*p* = 0.02).

Linear regression showed that younger age, gender, insurance type, being married and the households’ SES had significant effects on PHC trust level with R^2^ = 0.14383 (Table 3).

**Table 3-:** Correlation of various factors with PHC trust

Independent variables		Coef.	Std. Err.	Sig	[95% Conf. Interval]	
					Lower	Upper
Age		−.1374873	.0554154	0.013	−.2462224	−.0287522
Gender		−8.031263	1.432971	0.000	−10.84301	−5.219511
Married		−7.120119	2.039348	0.001	−11.12169	−3.118544
Insurance type^a^	No-Insurance	−21.69379	2.965023	0.000	− 27.51171	−15.87587
	Staff insurance	−18.90197	2.827646	0.000	−24.45033	−13.35361
	Social security	−19.63547	1.913221	0.000	−23.38956	−15.88138
	Military	−15.49104	4.777801	0.001	−24.86596	−6.116113
	Public insurance	−20.40934	2.815921	0.000	−25.9347	−14.8839
Diabetes		−2.257582	3.203666	0.481	−8.543762	4.028599
Depression		−4.900601	3.229798	0.129	−11.23806	1.43685
Hypertension		−3.24862	2.394783	0.175	−7.947623	1.450384
SES^b^	Middle level	4.144453	1.614019	0.010	.9774518	7.31145
	High level	4.897282	2.573098	0.05	−.1516085	9.946173

^a^Reference group: Rural insurance

^b^Reference group: Low level

## Discussion

PHC is a health delivery approach for optimizing equity in providing health services to the whole of community. The results of the present study showed that PHC trust was in an average level however, it was not acceptable. Age, gender, being married, type of health insurance, SES level, and living in the province capital were the affecting variables of PHC trust level. PHC focuses on population requirements and preferences, both individually and as a community, and systematically address health social determinants by providing proper services for health promotion [33]. PHC is the most comprehensive and primary level of health services delivery in Iran with the focus on deprived population [29, 34]. According to the literature, a trusted health team could provide patients with more emotional care and information and help them in shared decision making on their health (cognitive care) [33, 35, 36]. On the other side, low level of public trust in PHC will affect efficiency of health services and patients’ comply with healthy behaviors suggested by PHC providers [15, 37]. Kelley et al. (2014), in a systematic review of 13 randomized clinical trials, concluded that trust have a small but significant effect on health outcomes [17].

Tabrizi et al. reported the mean of public trust in health system as 53.91 ± 13.7 (out of 100) in Tabriz in the year 2014. Using a visual scoring method, people’s trust in urban health centers and public hospitals were calculated as 54.15 and 61 (out of 100), respectively [5]. Van der Schee et al. (2007) reported a mean public trust of 70 in the Netherlands [9], higher than our results. In a Chinese study, researchers revealed that the public trust in physicians experienced a significant decrease from 2011 to 2016 and they asked policy-makers for taking relevant actions [38]. PHC system needs to be more trustful than other sectors of health system. Because it provides preventive health services with the focus on population health requirements and interventions which support achieving Sustainable Development Goals (SDGs) and also address the social determinants of health such as healthy childhood. Identifying PHC trust determinants and employing proper policies to increase its level, must be a priority policy-making system.

Women, younger people, married people, people who have insurance, households with higher level of SES, and also participants from cities other than Tabriz (the province capital) showed higher level of trust with a significant difference (*p* < 0.05) when compared to corresponding variables. In contrast with our results, Zhao et al. (2017) resulted that younger people in China had lower level of trust in health system [39]. It was reported, in previous studies, that age had a positive and significant association with the level of trust saying that people with higher age have higher level of trust in health system or health providers [38, 40]. In fact, PHC system in Iran does not efficiently provide health services needed for middle-aged and elderly [34]. However, beside clinical services, individuals in these age groups mostly need routine care, education, and empowerment. Population aging is a real challenge in Iran and other countries. It makes PHC systems to consider the changing face of the health needs and adopt more responsibility and accountability. This could help to bring more trust on PHC system.

Results revealed that people with chronic disorders have less level of the PHC trust than others. This was consistent with Ronny et al. (2016) which stated that people with fewer chronic disorders had higher trust level on physicians [41]. This may be due to the prolonged treatment of these individuals and experiencing system deficiencies during their treatment. PHC system mostly provides care services and suggests lifestyle interventions for chronic patients which has long-term outcome. This is not a pleasant achievement for patients and they are seeking for short-term positive outcomes. This issue probably affects their trust level in PHC services.

It was revealed that demographic variables had a small but significant relation with PHC trust level (R^2^ = 0.14383). In a study by Jodyn E. Platt et al. (2017), demographics only described 18% of the trust in health system level [42]. Zhao et al. (2019) in study of trust in physicians trend in China in 2011–2016, revealed that demographic variables have a small share in predicting trust level [38]. Trust is a multifaceted concept which is affected by different social, economic, personal and behavioral variables. Trust in health system, as a complex system, is associated with institutional and individual factors and also the context and picture of health system in the society. Literature reported that interpersonal relation between providers and patients, health provider skills, patients’ experiences on the delivery and outcomes of health services, the quality of services provided by health facilities, and social image of health system made by social media affect public trust in health system [9, 43]. It was also reported that patients’ participation in PHC services delivery and shared decision making will lead to improved trust and public sphere of the health system [44, 45]. Policy-makers should adopt reforms on PHC system according to social context in order to create positive experiences. For countries, public trust in PHC is a vital prerequisite for increasing health level and also achieving Universal Health Coverage (UHC) goals. As a first level of health services delivery, PHC should be trustful to encourage people to employ healthy lifestyle and also to adopt healthy behaviors.

It should be in mind that PHC organization and services delivery model in Iran might influence the results. Moreover, use of cross-sectional design and self-reported questionnaire which did not provide casualty relations and may have been over or under-estimated can be other limitation. Notwithstanding, to the best of our knowledge, this is one of the first studies empirically explored PHC trust in a population and emphasized its importance in PHC policy development in the world.

## Conclusion

PHC trust needs to be in the focus range of all policies to reform health system. PHC trust level is a sight of health services quality and patients’ experiences. Public trust in PHC is not only influenced by individuals’ variables and experiences but also by health system and health providers’ characteristics and public sphere about PHC in the society. It is needed to work empirically more on PHC trust concept to identify building blocks of trust in and out of health system. To the best of our knowledge, this is one of the first studies providing a clear picture of public trust in PHC system in Iran. The results can guide policy-makers on determinants and give them clues to draw up proper reform policies affecting trust level. Moreover, PHC trust level could be used as a public indicator in health systems especially in Low and Middle Income Countries (LMIC), to lead in to system strengthening policies at the national and international levels.

## Data Availability

The datasets generated and analyzed during the current study are not publicly available due to the ethical restrictions made by the ethical committee but are available from the corresponding author on reasonable request.

## Abbreviations

PHC: Primary Health Care
UHC: Universal Health Coverage
